# Assessing the Anti-Inflammatory Effects of an Orally Dosed Enzymatically Liberated Fish Oil in a House Dust Model of Allergic Asthma

**DOI:** 10.3390/biomedicines10102574

**Published:** 2022-10-14

**Authors:** Crawford Currie, Bomi Framroze, Dave Singh, Simon Lea, Christian Bjerknes, Erland Hermansen

**Affiliations:** 1 Hofseth BioCare, Kipervikgata 13, 6003 Ålesund, Norway; 2 Division of Infection, Immunity and Respiratory Medicine, School of Biological Sciences, Faculty of Biology, Medicine and Health, Manchester Academic Health Science Centre, The University of Manchester, Manchester M13 9PL, UK; 3 The Medicines Evaluation Unit, Manchester University NHS Foundation Trust, Manchester M23 9QZ, UK; 4 Department of Clinical Medicine, University of Bergen, 5007 Bergen, Norway

**Keywords:** allergy, asthma, eosinophils, immune health, natural therapeutics

## Abstract

Eosinophils are a major driver of inflammation in a number of human diseases, including asthma. Biologic therapies targeting IL-5 have enabled better control of severe eosinophilic asthma, but no such advances have been made for enhancing the control of moderate asthma. However, a number of moderate asthma sufferers remain troubled by unresolved symptoms, treatment side effects, or both. OmeGo, an enzymatically liberated fish oil, has demonstrated antioxidant and anti-inflammatory properties including the reduction of eosinophilia. A house dust mite model of induced asthma in mice was utilized in this study, and OmeGo showed a significant reduction in eosinophilic lung and systemic inflammation and reduced lung remodelling compared to cod liver oil. The CRTH2 antagonist fevipiprant showed an anti-inflammatory profile similar to that of OmeGo. OmeGo has the potential to be a pragmatic, cost-effective co-treatment for less severe forms of eosinophilic asthma. Proof-of-concept studies are planned.

## 1. Introduction 

Eosinophils are well recognised as important drivers of a number of inflammatory diseases in humans, including asthma. Biologic therapies targeting cytokines involved in the activation of eosinophils have been a major advance in the management of eosinophilic asthma [1]; however, for milder forms of asthma, there have been no new additions to the treatment armamentarium for a number of years.

Polyunsaturated fatty acids (PUFAs) contained in the oil fraction of fresh fish provide anti-inflammatory and antioxidant benefits important for the maintenance of good health [2]. Observational and epidemiological studies indicate that regular fish consumption can have wide ranging benefits on human health, including the cardiovascular and respiratory systems. Indeed, changing dietary habits have seen a decline in fish consumption and an increase in the prevalence of asthma and allergic disease in Western countries [3,4,5]. To compensate for this, dietary supplementation with omega-3 fish oil is frequently used to try to attain the health benefits of consuming fresh fish. Fish oil PUFAs are known to be metabolized into specialized pro-resolving mediators (SPMs), which provide broad inflammation-resolving effects [6]. Whilst trials of omega-3 supplementation suggest a potential to reduce airway inflammation and improve lung function, the effects have been variable [7,8,9,10]. However, there is evidence that whole fish consumption during pregnancy and in young children can reduce the risk of developing allergic conditions, a benefit likely derived from the range of anti-inflammatory factors naturally contained in fish oil and not just omega-3 [11].

The potential to reduce the burden of asthma via a dietary intervention could be beneficial for sufferers and healthcare systems alike. This trial, therefore, assessed the extent to which oral OmeGo, an intervention more closely related to eating whole fish, could reduce lung inflammation in a standard animal model of induced asthma. OmeGo is minimally processed whole fish oil with low levels of oxidation and free fatty acids and contains all of the polyunsaturated acids found in whole fish, not just omega-3. Previous in vitro work demonstrated OmeGo to significantly reduce eosinophilic effector function, whereas this was not the case for oils containing either omega-3 alone or omega-3 and astaxanthin [12], and a similar comparative profile between OmeGo and omega-3 oils was also demonstrated in animal models of induced eosinophilia [13]. As these previous in vivo studies administered OmeGo by the intraperitoneal route, this paper describes experiments focused now on oral delivery, to provide information that is more relevant to its use in humans. Furthermore, we evaluated lung remodelling as well as inflammation to further assess the potential to improve health outcomes compared to omega-3 supplementation alone [14].

## 2. Materials and Methods

The study utilized an HDM model of asthma in mice to compare the impact of 7 days of treatment with OmeGo compared to either fevipiprant or cod liver oil on lung and serum inflammatory markers and lung fibrosis.

The study was conducted according to GLP guidelines and in accordance with the laws and regulations of India, where the studies were performed. The study was approved by the Institutional Animal Ethics Committee (proposal number 214429) before the start of the study. The health status of the animals was assessed by a veterinarian, and all were noted to be in good health. The animals were acclimated to the laboratory conditions, and randomisation to the five treatment groups occurred the day before the trial commenced. The five groups were: no treatment (negative control), 0.5 mL of cod liver oil (vehicle control), 18 mg or 32 mg of OmeGo (test item), or 2 mg fevipiprant (positive control), respectively.

**Figure 1:**
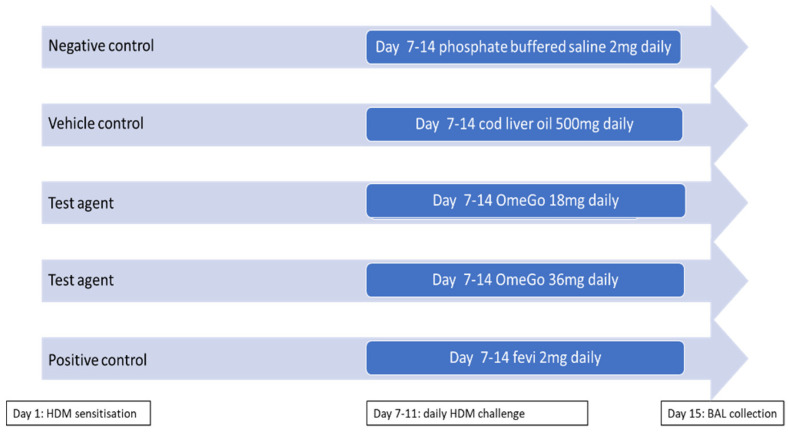
Experimental design. The mice were randomised into five groups of 10 animals per group. On day 1, animals were anaesthetised and sensitised intranasally with 1 µg HDM protein in 40 µL phosphate buffered saline (PBS). Following this, the mice were challenged daily with 10 µg HDM protein intranasally from day 7 to day 11. Oral interventions of PBS, cod liver oil, OmeGo low or high dose, or fevipiprant were given on days 7–14. BAL fluid was collected on day 15.

As per the experimental procedure shown in Figure 1, the study involved 50 healthy young adult female mice of 10 animals per group.

On day 1, the mice were anaesthetized using ketamine (100 mg/kg) and xylazine (10 mg/kg) given via intraperitoneal injection. HDM sensitisation was achieved with the intranasal application of 1 µg HDM protein in 40 µL phosphate buffered saline (PBS). Subsequently, daily intranasal HDM challenge was performed from day 7 to day 11 using 10 µg HDM protein in 40 µL PBS.

Between days 7 and 14, the mice received either cod liver oil, OmeGo, or fevipiprant treatment, all given orally. The fifth group of mice received no treatment (negative control). On day 15, the mice were anaesthetised and their trachea exposed to enable bronchoalveolar lavage (BAL) fluid collection using 3 mL of PBS containing 1 mM EDTA. The spleen was also removed from each animal, frozen and subsequently assessed for the extent of eosinophilia.

After collection, the BAL fluid was centrifuged (400× *g* at 4 °C for 7 min). The resulting cell pellet was collected, stored at −20 °C and subsequently analysed for total leucocyte cell count and differential cell count to provide eosinophil, neutrophil, lymphocyte and alveolar macrophage levels. A hemocytometer was used to measure total cell counts in the cell pellets and spleen tissue. For total leukocyte counts, the resuspended cell pellets in 50 µL phosphate buffered saline (PBS) were measured using a hemocytometer [15].

Serum HDM-specific IgE was assessed using the antigen-capture ELISA method [16]. Total lung collagen content was assessed using the calorimetric Quickzyme Total Assay Kit. Collagen content was normalized to the weight of each lung to be able to compare the total collagen value across groups.

Further details are contained in Appendix A. All analyses were performed in duplicate.

The animals were observed in the morning and evening to check for morbidity and mortality and were weighed during randomisation and on day 1, day 7 and day 15 of the study.

Necropsy at end of treatment was according to the guidelines of the CPSCEA committee.

The number of animals selected was guided by our previous HDM work, in which OmeGo was dosed intraperitoneally (IP) in five animals per group [12] and showed a significant 42% reduction in serum eosinophil count. To be conservative, in case of greater inter-animal responses with oral OmeGo compared to IP dosing, we chose to double the size to 10 animals per group in the present study. All raw data from this study were analysed using “Sigma Plot” v14 statistical software, and this was used to calculate the mean and standard deviations. All continuous data were checked for their homogeneity using the Shapiro–Wilk test. Once homogeneity was confirmed, the data were analysed using ANOVA, and data showing significance in their variances were subjected to unpaired *t*-test. *p* values ≤ 0.05 were deemed statistically significant.

## 3. Results

The initial analysis assessed vehicle control (cod liver oil) versus negative control. This showed non-significant, small to negligible numeric differences for total BAL leukocyte count, BAL eosinophil count and percent eosinophil in spleen tissue (*p* = 0.638, 0.382 and 0.314, respectively). All other analyses also showed minimal numeric differences between the two groups (vehicle and negative control). All efficacy analyses were, therefore, based in comparison to vehicle control, cod liver oil, rather than negative control.

At the end of the study, total leukocyte count in the BAL fluid showed a statistically significant decrease in the high-dose OmeGo (*p* < 0.05) and fevipiprant (*p* < 0.01) groups compared to cod liver oil. Analyses of individual cell counts, namely eosinophils, neutrophils, macrophages and lymphocytes, were undertaken and are described below.

None of the animals showed any treatment related ill-effects (morbidity or mortality), and there were no statistically significant differences in body weight in any of the treatment groups compared to the cod liver oil group (vehicle control). There was also no significant change in weight during the trial compared to baseline (day 1).

### 3.1. Cell Counts

#### 3.1.1. Eosinophils

**Figure 2:**
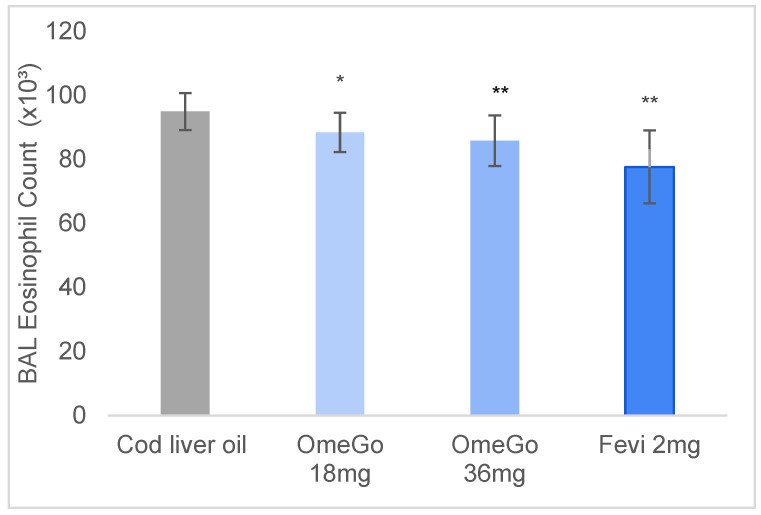
Eosinophil cell count in bronchoalveolar lavage (BAL) fluid of mice at study end, illustrating mean eosinophil count with cod liver oil (vehicle control), two doses of OmeGo, or fevipiprant. Cells were quantified with a hemocytometer. ** denotes *p* < 0.01, * denotes *p* < 0.05.

**Figure 3:**
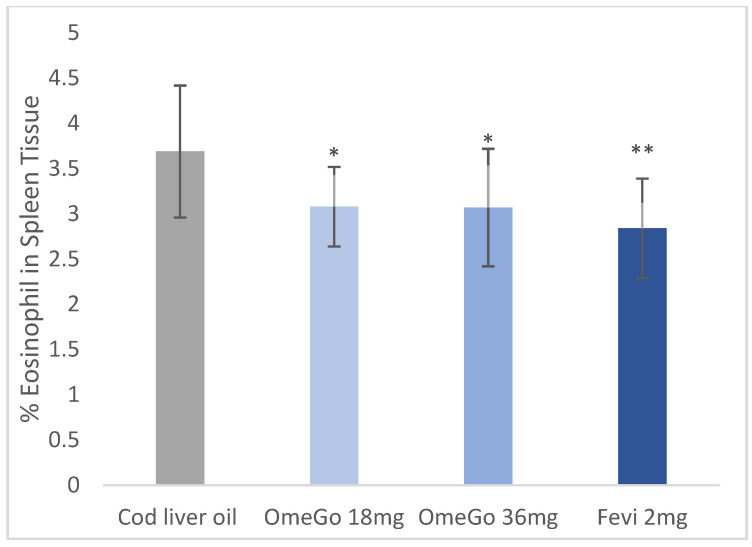
Percentage of eosinophils in spleen tissue of mice at study end with cod liver oil (vehicle control), two doses of OmeGo, or fevipiprant. Cells were quantified with a hemocytometer. ** denotes *p* < 0.01, * denotes *p* < 0.05.

The impact of OmeGo and fevipiprant on lung and splenic eosinophilia was assessed at the end of the trial. Compared to cod liver oil, a significant 7% (*p* ≤ 0.05) and 10% (*p* ≤ 0.01) decrease in BAL eosinophils was seen with low- and high-dose OmeGo, respectively, and an 18% (*p* ≤ 0.001) decrease with fevipiprant (Figure 2). Splenic eosinophilia was significantly reduced by 16% and 17% (both *p* ≤ 0.05) with low- and high-dose OmeGo, respectively, and by 23% (*p* ≤ 0.01) with fevipiprant (Figure 3).

#### 3.1.2. Neutrophils

**Figure 4:**
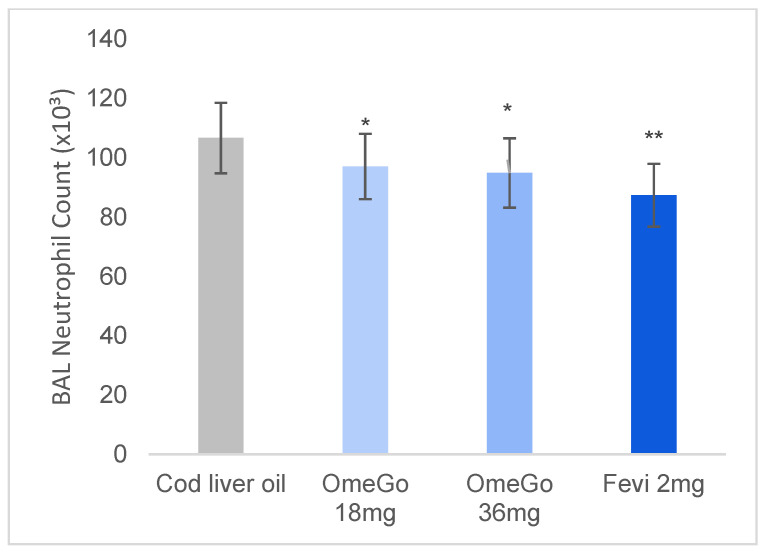
Cell count neutrophils in bronchoalveolar lavage (BAL) fluid of mice at end of study, illustrating mean neutrophil count with cod liver oil, two doses of OmeGo, or fevipiprant. Cells were quantified using a hemocytometer. *p* values less than 0.01 are summarized using two asterisks, and *p* values less than 0.05 are summarized with one asterisk.

By the end of the study, HDM sensitisation had resulted in higher lung neutrophils in the cod liver oil group compared to the OmeGo and fevipiprant groups. Low-dose and high-dose OmeGo reduced bronchoalveolar neutrophil count by 9% and 11% (both *p* ≤ 0.05), respectively, while in the fevipiprant group there was an 18% reduction (*p* = 0.01) versus cod liver oil (Figure 4).

#### 3.1.3. Macrophages and Lymphocytes

Unfortunately, the HDM sensitisation regimen employed in this study did not induce an increase in lymphocytes in the BAL fluid at study end, with no numeric difference noted between any of the groups, including negative control. OmeGo and fevipiprant showed a numeric reduction in alveolar macrophages of 14%, but this difference was not significant.

### 3.2. Cytokines

**Figure 5:**
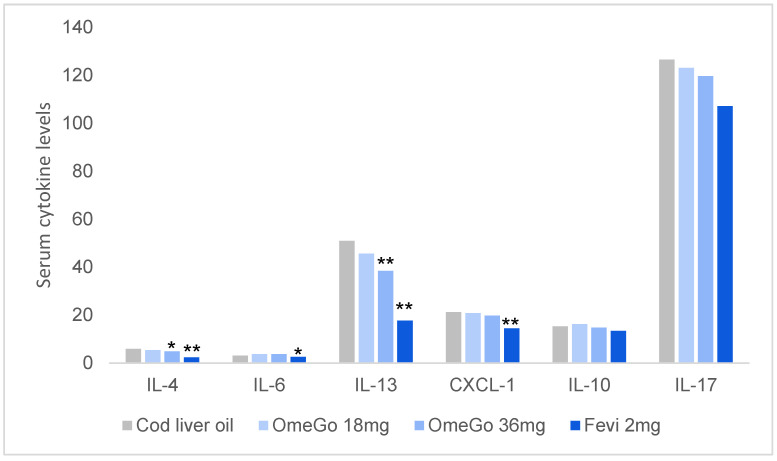
Mean serum cytokine levels at study end with cod liver oil, two doses OmeGo, or fevipiprant. ** denotes *p* < 0.01, * denotes *p* < 0.05.

Low-dose OmeGo significantly reduced IL-13 by 11%, and high-dose OmeGo drove a 24% reduction in serum IL-13 levels (*p* < 0.01) and a 17% reduction in IL-4 (*p* < 0.05), all compared to cod liver oil. OmeGo did not significantly impact IL-6, IL-17A or CXCL-1 levels. Fevipiprant significantly reduced CXCL-1 (32%, *p* < 0.001), IL-4 (58%, *p* < 0.001), IL-6 (18%, *p* < 0.05) and IL-13 (63%, *p* < 0.001) but not IL-10 or IL-17A. In terms of changes in serum IgE levels, the 12% reduction with OmeGo and the 6% reduction with fevipiprant were non-significant (Figure 5).

### 3.3. Lung Collagen Content

**Figure 6:**
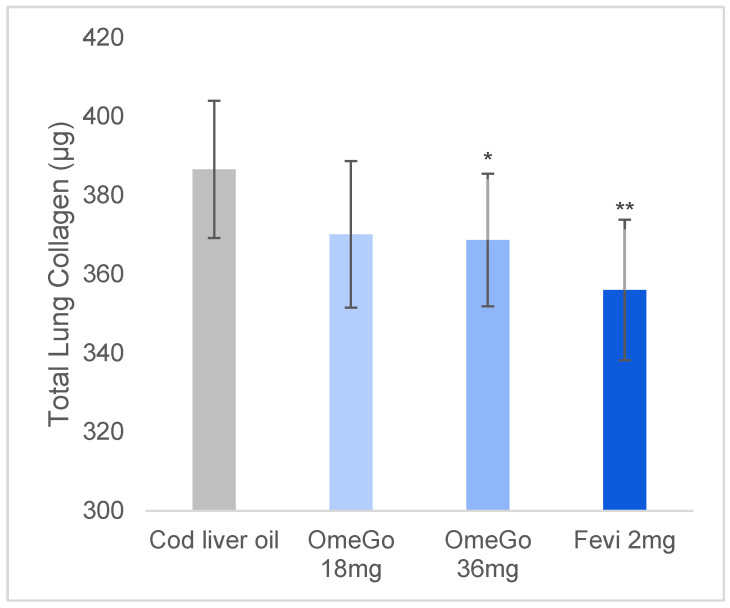
Mean total lung collagen content at the end of the study with cod liver oil, two doses of OmeGo, or fevipiprant, assessed using the calorimetric Quickzyme Total Assay Kit. ** denotes *p* < 0.01, * denotes *p* < 0.05.

Total lung collagen content was significantly reduced by OmeGo and fevipiprant compared to vehicle control: a 4% and 5% reduction, respectively, with low- and high-dose OmeGo (*p* < 0.05) and an 11% reduction with fevipiprant (*p* < 0.01) at study end (Figure 6). No other histopathological assessments of the lung were undertaken.

## 4. Discussion

In this study, oral OmeGo significantly reduced lung and splenic eosinophilia compared to cod liver oil (vehicle control) in a house dust mite (HDM) mouse model of asthma. These results are consistent with previous work that assessed OmeGo’s modulation of eosinophil function, including in vivo studies with OmeGo dosed via intraperitoneal injection [12] and in vitro work in human eosinophils [13].

This study further characterised OmeGo’s modulation of inflammatory mediators relevant to asthma pathophysiology. Beyond eosinophil modulation, oral OmeGo significantly reduced BAL (bronchoalveolar lavage) neutrophil levels and total lung collagen content. Airway remodelling is a typical feature in persistent asthma, contributing to airflow limitation. Collagen deposition occurs early in the natural history of asthma [17,18] and is correlated with disease severity [19]. Allergen exposure causes an increase in airway remodelling markers in patients with asthma [20], with IL-13 an underlying driver of the process. Thus, OmeGo’s significant impact on IL-13 provides biologic plausibility to this initial result in assessing OmeGo’s potential to reduce lung remodelling [21].

IL-4 is an important driver in the initiation of lung inflammation in asthma, including Th2 cell proliferation and IgE synthesis [22,23], and high-dose OmeGo significantly reduced IL-4 by 17% (*p* < 0.05); however, the numeric reduction in serum IgE did not quite achieve significance.

The house dust mite is a common air-borne allergen, with up to 85% of asthma patients being allergic to HDM [24]. The HDM model of induced asthma is, therefore, commonly used to mimic the inflammatory and allergic milieu found in many asthma patients, namely eosinophilia, raised IgE levels and other inflammatory mediators associated with Type 2 inflammation as well as neutrophilia [24,25]. Consistent with the published literature, HDM sensitisation in our study resulted in a leukocytosis driven by eosinophils and neutrophils. We saw no impact on lymphocyte numbers and only a limited impact on macrophage numbers, and previous work indicates that longer duration HDM models are more likely to induce a significant expansion of macrophage numbers [26] and drive greater fold increases overall in leukocyte recruitment [27,28].

A 5-week HDM mouse model of induced asthma demonstrated a 500-fold increase in BAL eosinophils, equating to 1.5 million eosinophils/mL on flow cytometry [27]. In contrast, a short duration, higher HDM dose (total of 300 µg) in vivo study resulted in a BAL eosinophilia of around 350,000 cells/mL in the saline control group, which was almost totally resolved with intraperitoneal dexamethasone and reduced by approximately 50% with CRTH2 antagonist treatment [29]. We utilized a standard short-exposure HDM model protocol with a total HDM challenge dose of 50 µg; this resulted in an eosinophil count of 97,400 cells/mL in the negative control group (saline). This lower level of induced leukocytosis may explain the smaller magnitude of cell count reductions with CRTH2 antagonism (fevipiprant) in our study, such as a 20% decrease in eosinophilia, compared to previous ex vivo studies with CRTH2 antagonists. This methodological difference may also have influenced the magnitude of effect observed with OmeGo.

The CRTH2 receptor is a well recognised activator of eosinophil-driven inflammation [30,31], hence our choice of the CRTH2 antagonist (fevipiprant) as a positive control. The effects of fevipiprant and OmeGo were generally similar with regard to lung and systemic inflammation, with significant reductions in both eosinophil and neutrophil counts as well as total lung collagen. CRTH2 antagonists are known to inhibit experimental allergen challenge responses in humans [32,33], and the similarity of the effects observed in this in vivo animal model indicates potential for OmeGo to attenuate allergic responses in humans.

Vehicle control, consisting of 0.5 mL cod liver oil, containing DHA (docosahexanoic acid) and EPA (eicosapentaenoic acid) omega-3, did not show an effect on any of the endpoints compared to negative control. This contrasting effect between OmeGo and cod liver oil is consistent with our previous work on the modulation of eosinophil function with OmeGo. This effect appears to be driven by a bioactive fraction not present in highly processed supplements [12,13]. The variable outcomes seen with omega-3 supplementation compared to the consumption of fresh fish [10] suggests that minimal processing helps to retain the full bioactivity of the individual PUFAs to provide health benefits associated with eating fresh fish.

Our study has a number of limitations. By using a shorter-duration HDM exposure model, we did not elicit an elevation in lymphocytes and only a moderate increase in macrophage count, which, therefore, limits the insights on the potential of the interventions to modulate the activity of these immune cells. In addition, analyzing other mediators of inflammation would have been valuable, but there was insufficient serum to analyse changes in IL-5, a cytokine involved in eosinophil activation and recruitment. The elevation of IL-17 was not modulated by OmeGo, and other analyses for inflammatory mediators such as the impact on IL-1β and granulocyte-macrophage colony-stimulating factor (GM-CSF) in the BAL could have provided useful insights into OmeGo’s impact on lung barrier function and provided mechanistic explanations for the modulation of lung collagen deposition. Investigation of primary inflammatory pathway mediators, such as nuclear factor kappa B (NFκB), GATA-3, and peroxisome proliferator-activated receptor gamma (PPARγ), would have been valuable to assess the means by which OmeGo reduced lung inflammation. Additionally, further immunohistochemistry work, including assessments of lung damage and collagen deposition, could provide more insights regarding pharmacological effects. Nevertheless, the study builds on previous work and elucidates further the anti-inflammatory action of the whole fish oil, OmeGo.

## 5. Conclusions

OmeGo, dosed orally, significantly reduced lung and systemic inflammation in an HDM mouse model of induced asthma. Reductions in eosinophils and neutrophils were accompanied by a reduction in total lung collagen, suggesting the potential to moderate airway inflammation and remodelling and help maintain lung function. Human studies are planned in subjects with mild to moderate asthma to assess whether these results can translate into improved asthma control with OmeGo added to standard-of-care treatment.

## Data Availability

The raw data, original Project Plan, original final report and a sample of the test items are retained in the Archives of sa-FORD (Sanctuary for Research and Development). Plot number V-10, MIDC Industrial Area, Taloja, Dist: Raigad 410 208, India for a period of 5 years.

## Appendix A

The animals were allocated to different test groups by a manual randomization method. Individual body weights of allocated animals were within 20% of the group means, and the body weights of the animals were analysed statistically to rule out significant differences between the groups before administration of the HDM extract. Following allocation to the experimental groups, each animal was uniquely identified by turmeric fur marking/micro-toe pad tattooing and colour-coded cage card labelled with project number, project type, test system, sex, dose, group, animal number, date of treatment, and experiment start and completion dates.

The mice were housed in groups of five animals per individually ventilated cages. Corn cob, prepared from pure corn and dried, freed of dust and sterilized, was procured from an approved vendor and used as bedding material. The bedding was replaced as often as necessary to keep the animals and their surroundings clean and dry. The room temperature was maintained between 21 °C to 22.5 °C and relative humidity at 50.9% to 66.8% for the duration of the study. Artificial light was set to give a cycle of 12 h light and 12 h dark. Adequately filtered air was provided with at least 12 changes per hour. The animals were offered a conventional laboratory rodent diet ad libitum supplied by Nutrivet Life Sciences, and Aquaguard filtered drinking water was provided ad libitum.

For the differential leukocyte counts, including eosinophil count, 80 µL of resuspended cell pellets were stained using the HEMA3 stain set (Fisher Scientific), and centrifuged using a StatSpin Cytofuge 2 (Beckman Coulter).

Ninety-six well plates coated with 5 µg HDM in 100 µL coating buffer and incubated for 12 h at 4 °C were blocked with 200 µL/well of assay diluent. Precleared serum samples (Protein G Sepharose beads) were washed with PBS, and a 50 µL aliquot of undiluted serum was added to each well and incubated at 4 °C overnight. The wells were washed with PBS; 100 µL of biotin-anti-mouse IgE (Sigma, Virginia Beach, VA, USA) was added and incubated for 1 h, followed by a 30 min incubation with avidin-horse radish peroxidase. TMB substrate solution (100 µL) was added to each well and incubated in the dark for 30 min. The reaction was stopped with 2 N sulphuric acid. Optical densities, normalized to saline controls, were read at 450 nm using a Varian Cary 50 spectrophotometer.
